# Predicting Survival Benefit of Sparing Sentinel Lymph Node Biopsy in Low-Risk Elderly Patients With Early Breast Cancer: A Population-Based Analysis

**DOI:** 10.3389/fonc.2020.01718

**Published:** 2020-09-11

**Authors:** Li Xu, Nan Wen, Juanjuan Qiu, Tao He, Qiuwen Tan, Jiqiao Yang, Zhenggui Du, Qing Lv

**Affiliations:** Department of Breast Surgery, West China Hospital, Sichuan University, Chengdu, China

**Keywords:** breast cancer, elderly, sentinel lymph node biopsy, prognosis, nomogram, SEER program

## Abstract

**Objective:** The application of sentinel lymph node biopsy (SLNB) in elderly patients with early breast cancer remains somewhat controversial. This study aimed to establish individualized nomograms to predict survival outcomes of elderly patients with and without SLNB and find out which patients could avoid SLNB.

**Methods:** A total of 39,962 ≥70-year-old patients diagnosed with T1–T2 breast cancer in 2010–2015 were included from the Surveillance, Epidemiology, and End Results (SEER) program and were divided into the training set (*n* = 29,971) and the validation set (*n* = 9,991). Axillary surgery was not specified in the SEER database, and we defined removing one to five lymph nodes as SLNB. Survival analysis was performed using the Kaplan–Meier plot and log-rank test. Multivariate Cox analysis was utilized to identify risk factors for overall survival (OS) and breast-cancer-specific survival (BCSS). Nomograms and a risk stratification model were constructed.

**Results:** In the training set, patients with SLNB had better OS (adjusted HR 0.57, *P* < 0.001) and BCSS (adjusted HR 0.55, *P* < 0.001) than patients without SLNB. Multivariate COX analysis identified age, marital status, grade, subtype, T stage, and radiation as independent risk factors for OS and BCSS in both SLNB and non-SLNB groups (all *P* < 0.05). They were subsequently incorporated to establish nomograms to predict 3- and 5-year OS and BCSS for patients with or without SLNB. The concordance index ranged from 0.687 to 0.820, and calibration curves in the internal set and external set all demonstrated sufficient accuracies and good predictive capabilities. Further, we generated a risk stratification model which indicated that SLNB improved OS and BCSS in high-risk group (OS: HR 0.49, *P* < 0.001; BCSS: HR 0.54, *P* < 0.001), but not in the low-risk group (all *P* > 0.05).

**Conclusion:** Well-validated nomograms and a risk stratification model were constructed to evaluate survival benefit from SLNB in elderly patients with early-stage breast cancer. SLNB was important for patients in the high-risk group but could be omitted in the low-risk group without sacrificing survival. This study could assist clinicians and elderly patients to weigh the risk–benefit of SLNB and make individualized decisions. We look forward to more powerful evidence from prospective trials.

## Introduction

The concept of the treatment strategy for breast cancer has shifted from “maximum tolerated therapy” to “minimum effective therapy.” For breast cancer patients, axillary staging is an important part of the surgical process, and now sentinel lymph node (SLN) biopsy (SLNB), with satisfactory sensitivity and accuracy, has been established as the standard care for patients with early-stage breast cancer (1). SLNB plays an important role not only in guiding decisions regarding postoperative adjuvant treatment but also in reducing tumor burden of axillary nodes. A series of studies have clarified that unnecessary axillary lymph node dissection (ALND) can be spared without sacrificing survival in patients with clinically negative nodes, micrometastatic SLNs, or even limited macrometastatic SLNs, especially for SLN-micrometastatic or SLN-macrometastatic patients with breast conservative surgery but not for those with total mastectomy (2). Some clinical trials are also focusing on SLNB as an alternative to ALND in the setting of neoadjuvant chemotherapy (2).

However, the role of SLNB in elderly patients with low-risk breast cancer is yet controversial. Although SLNB is associated with improved quality of life and reduced morbidities compared with ALND (3, 4), a considerable number of patients undergoing SLNB still suffer from arm and shoulder complications, which could be more severe in elderly patients (5, 6). SLNB using blue dye may also cause potential adverse effects including anaphylaxis (7). And nowadays, adjuvant therapy depends more on tumor's biological characteristics than the results of SLNB. With improvements in multimodality therapy, the tumor burden can be further diminished by nonsurgical treatments including advanced radiotherapy, optimized chemotherapy, and anti-HER2 therapy regimens, novel agents for endocrine therapy and immunotherapy. Additionally, elderly patients with comorbidities may not gain survival benefit from complete axillary staging (8). For elderly patients who often face multiple comorbidities and have a lower tolerance of aggressive treatment than the younger patients, the risk of SLNB must be balanced with the benefit of staging and local control. While earlier studies suggested that SLNB should be offered in elderly patients (9–11), several recent studies indicated that elderly patients with early-stage and hormone-positive breast cancer gained limited survival benefit from SLNB resulting in an increasing omission of SLNB in appropriately selected patients (12–14). Several ongoing prospective trials, such as the Sentinel node vs. Observation after axillary Ultra-SouND (SOUND) and the Intergroup-Sentinel-Mamma (INSEMA), are evaluating whether SLNB can even be avoided in early breast cancer patients with a negative preoperative axillary assessment (including axillary ultrasound exploration and biopsy for suspicious lymph nodes) (15, 16). Heretofore, there is a paucity of literature regarding omission of SLNB in elderly women with low-risk breast cancer, and few guidelines have explicitly stated the application of SLNB in the elderly.

This study aimed to determine whether elderly patients with primary T1–T2 breast cancer can benefit from SLNB based on the data from the Surveillance, Epidemiology, and End Results (SEER) program. Individual nomograms were subsequently established to predict 3- and 5-year survival of this population with or without SLNB by taking multiple risk factors into consideration, and a risk stratification model was utilized to determine which patients can omit SLNB safely.

## Materials and Methods

### Database and Patient Selection

This retrospective study extracted data from the SEER program, which collects the information of cancer patients of 18 cancer registries and covers ~27.8% of the US population (17). The SEER database 8.3.6 was queried for ≥70-year-old women who were diagnosed with T1–T2 (American Joint Committee on Cancer seventh edition, AJCC T, 7th ed) primary breast cancer as their only cancer or first of subsequent cancers from 2010 to 2015. This study was reviewed and approved by the institutional review board of our institution, and informed consent was exempt because all records of the database are anonymous and open to the public.

Variables involved in this study were demographic characteristics (age at diagnosis, race, marital status, and benign or borderline tumor history), disease characteristics [lateral, tumor location, histology, grade, subtype (ER, PR, and HER2 status), and T and N stage], treatment characteristics (breast surgery type, chemotherapy, and radiotherapy), and survival status (survival time and cause of death). Age at diagnosis was transformed into categorical variables (70–74, 75–79, 80–84, and ≥85). Based on the code information in SEER, we divided tumor location into three groups (outer quadrant and axillary tail, inner quadrant, and others). Histology was categorized into five groups according to the latest classification of the World Health Organization (18): no special type (NST); invasive lobular carcinoma (ILC); favorable (tubular/mucinous/papillary); metaplastic; and others (histological types other than the above four types).

The “number of regional lymph nodes examined” code was used to distinguish between SLNB and non-SLNB. The SEER database does not specify the type of axillary surgery, so based on the AJCC definition of a standard ALND (at least six lymph nodes removed) (19), we defined that one to five lymph nodes examined were categorized as the SLNB group. No nodes examination or only aspiration of regional nodes was classified into the non-SLNB group. Patients with six or more nodes examined were categorized as ALND, and patients with an unknown number of nodes examined were excluded.

**Figure 1:**
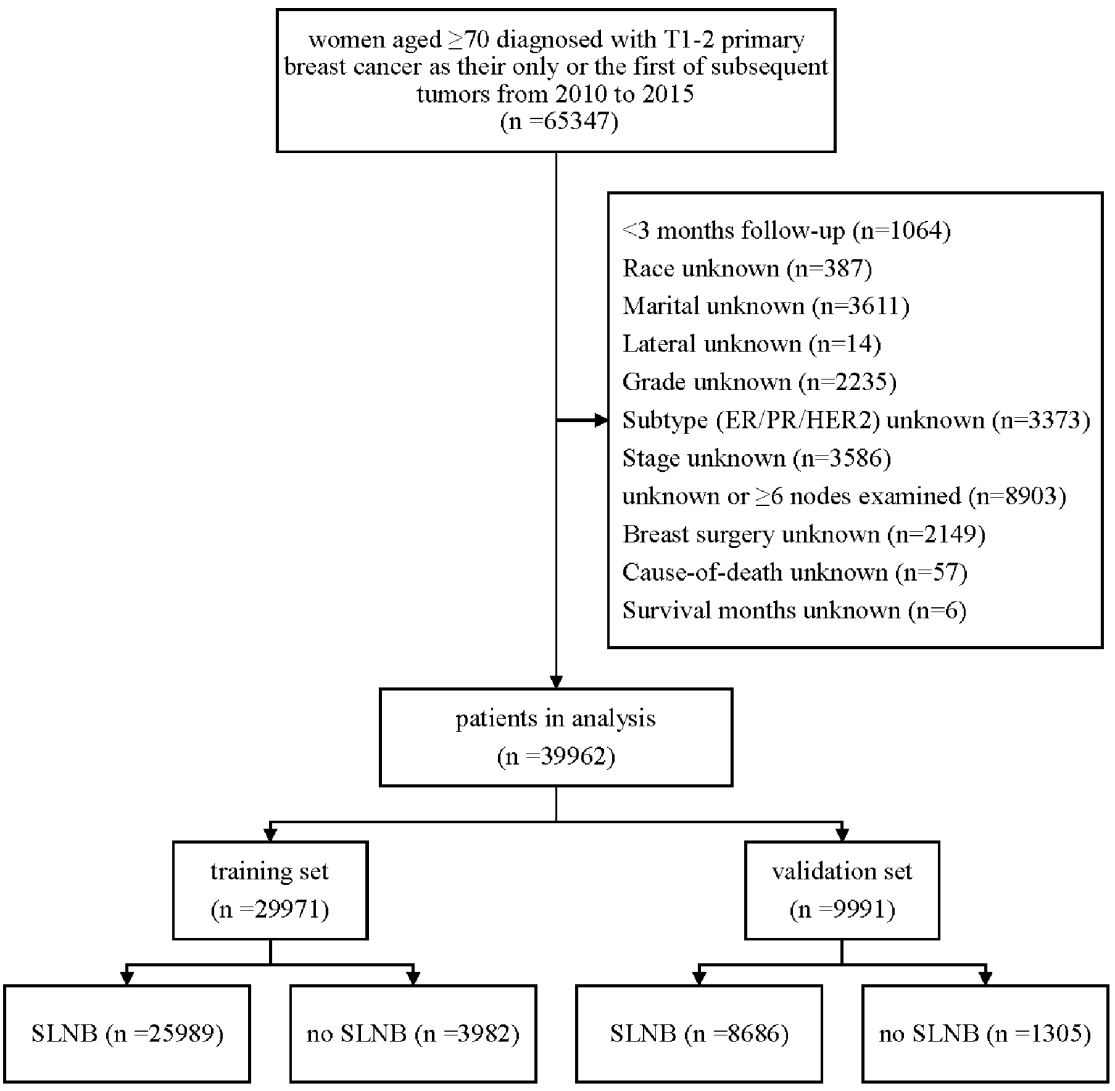
Flow chart of data selection.

The detailed exclusion criteria are illustrated in Figure 1. Briefly, patients with <3 months' survival or unknown follow-up were excluded to minimize immortal time bias. Subsequently, we dropped patients with unknown or unspecified variable's information.

### Statistical Analysis

All eligible patients were randomly assigned 3:1 into two cohorts: the training set for survival analysis and construction of nomograms and the validation set for external validation. Categorical characteristics were compared using Pearson chi-square test, and continuous characteristics were compared using Student *t*-test.

The outcomes of this study were breast-cancer-specific survival (BCSS) and overall survival (OS). BCSS was defined as the time from the date of diagnosis to the date of death attributed to breast cancer using cause-specific death classification in the SEER database. OS was defined as the time from the date of diagnosis to death due to any causes. The Kaplan–Meier plot and log-rank test were utilized to compare OS and BCSS between different groups. Univariate and multivariate COX proportional hazards regression models were utilized to detect independent risk factors. Subsequently, subgroup analysis was utilized to evaluate the survival benefit of SLNB in each subgroup.

For survival analysis in cohorts with and without SLNB, all variables with *P*-value <0.05 in univariate Cox analysis were included in multivariate Cox analysis. Four nomograms were developed to estimate 3- or 5-year OS and BCSS for patients with or without SLNB by involving risk factors according to the result of multivariate Cox analysis. Internal validation in the training set and external validation in the validation set were performed to evaluate the accuracy of these nomograms by a bootstrap validation method with 1,000 resamples. The concordance index (C-index) was applied to measure the discrimination of the model. The consistency between the actual observed outcome and the nomogram predicted survival probability was estimated by calibration curves.

Analyses were conducted by Stata/MP version 16.0 (StataCorp LP, College Station, TX) and the packages (rms, hmisc, survival, etc.) in R software version 3.6.1 (http://www.r-project.org). Statistical significance was determined with a two-tailed *P* < 0.05.

## Results

### Characteristics of Eligible Patients

After rigorous screening and selection (Figure 1), a total of 39,962 eligible patients were randomly assigned into the training set (*n* = 29,971) and the validation set (*n* = 9,991). In the training set and validation set (Supplementary Table 1), the distribution of baseline features was similar in both sets, and no significant difference was found between the two cohorts except N stage, implying a high value of external validation. Among the 29,971 patients in the training set, 85% (26,010/29,971) patients received SLNB, and 15% (3,961/29,971) did not. The clinicopathological differences in patients with and without SLNB in the training set were shown in Table 1. Of note, the proportion of patients without SLNB increased with age, and they tended to have breast cancer with positive hormone receptor or good differentiation. Meanwhile, the proportions of mastectomy, chemotherapy, and radiotherapy in the non-SLNB group were lower than that in the SLNB group (all *P* < 0.05), possibly because older people patients are often in poor general health and suffer from many complications.

**Table 1:** Demographic and disease characteristics of patient in training set.

**Variables**	**No SLNB (*n* = 3,982) n (%)**	**SLNB (*n* = 26,089) *n* (%)**	***p*-value**
**Age at diagnosis, y**			<0.001
70–74	546 (13.7)	11,571 (44.5)	
75–79	707 (17.8)	7,778 (29.9)	
80–84	1,060 (26.6)	4,462 (17.2)	
85+	1,669 (41.9)	2,178 (8.4)	
**Race**			0.176
White	3,440 (86.4)	22,150 (85.2)	
Black	291 (7.3)	1,961 (7.5)	
AIA	14 (0.4)	107 (0.4)	
API	237 (6.0)	1,771 (6.8)	
**Marital**			<0.001
Unmarried	2,674 (67.2)	13,312 (51.2)	
Married	1,308 (32.8)	12,677 (48.8)	
**Benign or borderline tumors history**			1.000
No	3,955 (99.3)	25,814 (99.3)	
Yes	27 (0.7)	175 (0.7)	
**Lateral**			0.600
Right	1,944 (48.8)	12,568 (48.4)	
Left	2,038 (51.2)	13,421 (51.6)	
**Tumor location**			0.003
Outer quadrant	1,586 (39.8)	10,961 (42.2)	
Inner quadrant	826 (20.7)	5,484 (21.1)	
Others^a^	1,570 (39.4)	9,544 (36.7)	
**Histology (ICD-O-3)^b^**			<0.001
NST	3,238 (81.3)	21,620 (83.2)	
ILC	343 (8.6)	2,746 (10.6)	
Favorable	274 (6.9)	1,126 (4.3)	
Metaplastic	21 (0.5)	128 (0.5)	
Others	106 (2.7)	369 (1.4)	
**Grade**			<0.001
Well; I	1,415 (35.5)	8,145 (31.3)	
Moderately; II	1,868 (46.9)	12,414 (47.8)	
Poorly; Grade III/IV	699 (17.6)	5,430 (20.9)	
**Subtype**			0.005
HR+/HER2–	3,395 (85.3)	21,600 (83.1)	
HR+/HER2+	233 (5.9)	1,646 (6.3)	
HR–/HER2–	273 (6.9)	2,146 (8.3)	
HR–/HER2+	81 (2.0)	597 (2.3)	
**ER**			0.003
Negative	381 (9.6)	2,901 (11.2)	
Positive	3,601 (90.4)	23,088 (88.8)	
**PR**			0.021
Negative	791 (19.9)	5,584 (21.5)	
Positive	3,191 (80.1)	20,405 (78.5)	
**HER2**			0.124
Negative	3,668 (92.1)	23,746 (91.4)	
Positive	314 (7.9)	2,243 (8.6)	
**Stage, AJCC 7th**			0.070
I	2,816 (70.7)	18,743 (72.1)	
II	1,166 (29.3)	7,246 (27.9)	
**T**			<0.001
T1	2,828 (71.0)	19,712 (75.8)	
T2	1,154 (29.0)	6,277 (24.2)	
**N**			<0.001
N0	3,938 (98.9)	23,173 (89.2)	
N1	44 (1.1)	2,816 (10.8)	
**Breast surgery type**			<0.001
BCS	3,477 (87.3)	19,517 (75.1)	
Mastectomy	505 (12.7)	6,472 (24.9)	
**Radiation**			<0.001
No/unknown	2,913 (73.2)	11,935 (45.9)	
Yes	1,069 (26.8)	14,054 (54.1)	
**Chemotherapy**			<0.001
No/unknown	3,851 (96.7)	22,817 (87.8)	
Yes	131 (3.3)	3,172 (12.2)	
**Follow-up months, mean(SD)**	37.39 (20.12)	41.98 (20.72)	<0.001

*AIA, American Indian/Alaska Native; API, Asian or Pacific Islander; NST, no special type; ILC, invasive lobular carcinoma; SLNB, sentinel lymph node biopsy; BCS, Breast reserving mastectomy/Lumpectomy*.

a *“others” includes “Central portion of breast,” “Breast includes Nipple” and “Overlapping lesion of breast such as 3, 6, 9, 12 o'clock” as recorded in the SEER database*.

b *“favorable” includes tubular, mucinous and papillary; “Others” means histological types other than above four types*.

### Analysis of Survival Benefits From Sentinel Lymph Node Biopsy

**Figure 2:**
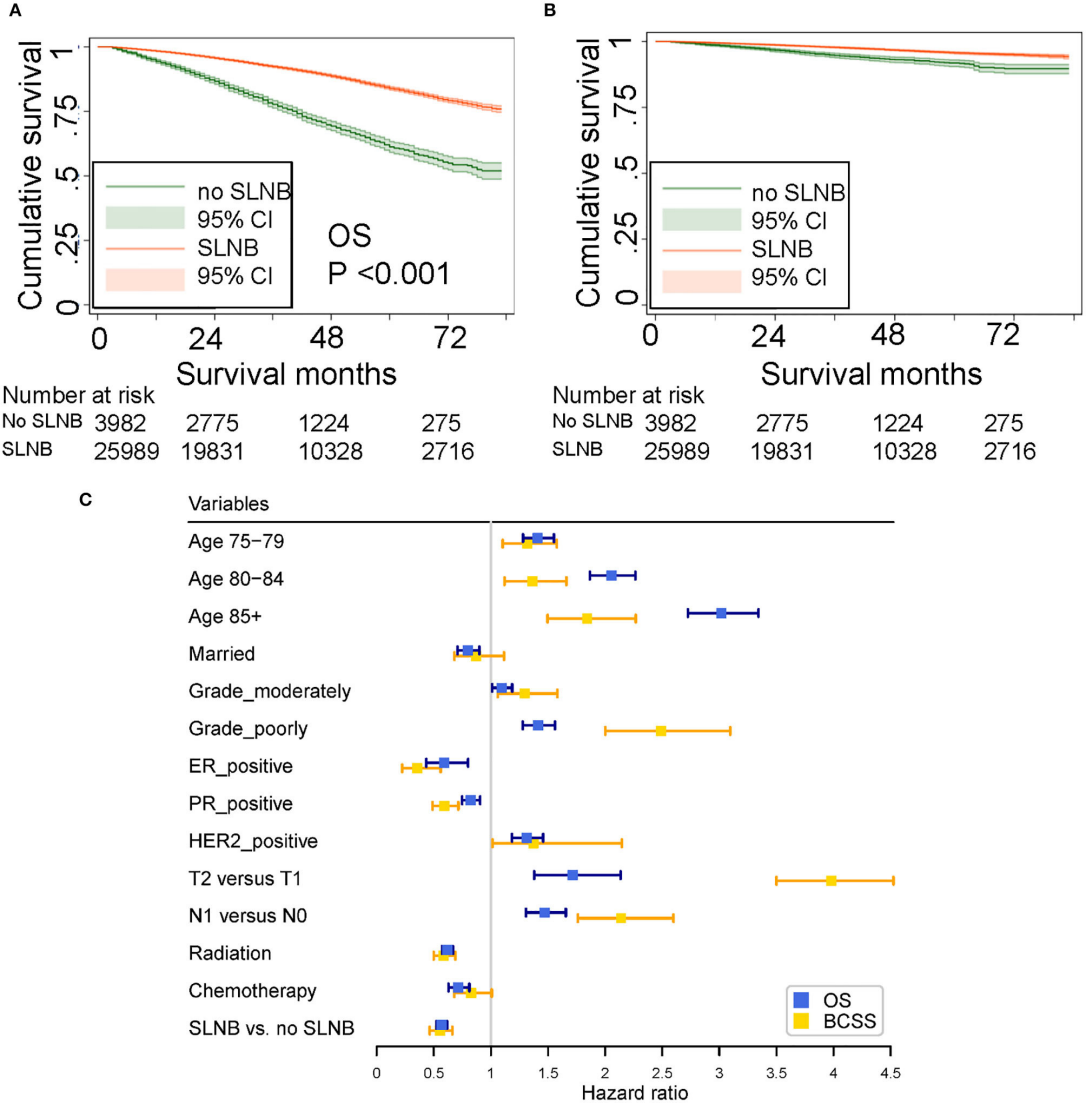
**(A)** Unadjusted overall survival (OS). **(B)** Unadjusted breast cancer-specific survival (BCSS) curves plotted by the Kaplan–Meier method for patients who received and did not receive SLNB. **(C)** Forest plot showing the results of multivariate COX analysis for OS and BCSS.

In the training set (*n* = 29,971), median follow-up time was 39 months (mean, 41.37 months; range, 3–83 months). At the time of the last follow-up, 3,952 patients were dead, 941 of which were dead directly from breast cancer. As shown in the Kaplan–Meier plot (Figures 2A,B), patients with SLNB had better OS (SLNB vs. no SLNB: unadjusted HR 0.35; 95% CI, 0.32–0.37; *P* < 0.001) and BCSS (SLNB vs. no SLNB: unadjusted HR 0.46; 95% CI, 0.39–0.53; *P* < 0.001) than patients without SLNB. Considering that the baselines of the SLNB and non-SLNB groups are inconsistent due to the retrospective nature of this study, we performed univariate and multivariate COX analyses to adjust potential confounding factors (Supplementary Tables 2, 3), and SLNB still exerted a protective factor (SLNB vs. no SLNB: adjusted HR of OS 0.57; 95% CI, 0.52–0.62; adjusted HR of BCSS 0.55; 95% CI, 0.46–0.66; all *P* < 0.001) as shown in the forest plot (Figure 2C). In order to determine if elderly patients in a specific subgroup could benefit from SLNB, subgroup analysis stratified by clinicopathological characteristics were performed (Table 2). The results of multivariate Cox analysis showed that SLNB exerted a significant survival benefit on both OS and BCSS in most of the subgroups (*P* < 0.05).

**Table 2:** Subgroup analysis of OS and BCSS outcomes.

**Variables**	**OS**		**BCSS**	
	**HR (95% CI)**	***P*-value**	**HR (95% CI)**	***P*-value**
**Age at diagnosis, y**				
70–74	0.447 (0.350–0.571)	<0.001	0.559 (0.397–1.353)	0.955
75–79	0.508 (0.420–0.616)	<0.001	0.492 (0.336–0.719)	<0.001
80–84	0.621 (0.535–0.720)	<0.001	0.559 (0.397–0.787)	0.001
85+	0.593 (0.526–0.669)	<0.001	0.529 (0.402–0.696)	<0.001
**Race**				
White	0.555 (0.509–0.605)	<0.001	0.541 (0.450–0.651)	<0.001
Black	0.695 (0.525–0.921)	<0.011	0.653 (0.382–1.120)	0.194
AIA	0.221 (0.081–0.603)	0.003	0.290 (0.056–1.149)	0.034
API	0.587 (0.393–0.875)	0.009	0.392 (0.193–0.793)	0.009
**Marital**				
Unmarried	0.460 (0.355–0.597)	<0.001	0.485 (0.393–0.599)	0.004
Married	0.579 (0.499–0.672)	<0.001	0.669 (0.478–1.035)	0.127
**Benign or borderline tumors history**				
Yes	0.528 (0.202–1.383)	0.092	0.195 (0.036–1.051)	0.057
No	0.577 (0.533–0.624)	<0.001	0.554 (0.467–0.657)	<0.001
**Lateral**				
Right	0.577 (0.516–0.646)	<0.001	0.594 (0.465–0.759)	<0.001
Left	0.573 (0.513–0.639)	<0.001	0.518 (0.409–0.656)	<0.001
**Tumor location**				
Outer quadrant	0.588 (0.520–0.664)	<0.001	0.623 (0.476–0.816)	0.001
Inner quadrant	0.630 (0.522–0.760)	<0.001	0.998 (0.0.898–1.398)	0.075
Others^a^	0.542 (0.479–0.612)	<0.001	0.449 (0.347–0.581)	<0.001
**Histology (ICD-O-3)^b^**				
NST	0.594 (0.544–0.648)	<0.001	0.574 (0.477–0.691)	<0.001
ILC	0.462 (0.361–0.592)	<0.001	0.384 (0.225–0.654)	<0.001
Favorable	0.505 (0.366–0.697)	<0.001	0.400 (0.156–1.023)	0.056
Metaplastic	0.301 (0.145–0.624)	0.001	0.551 (0.186–1.625)	0.201
Others	0.526 (0.324–1.273)	<0.259	0.505 (0.123–1.790)	0.741
**Grade**				
Well; Grade I	0.603 (0.520–0.699)	<0.001	0.472 (0.311–0.717)	<0.001
Moderately; Grade II	0.585 (0.521–0.658)	<0.001	0.585 (0.443–0.772)	<0.001
Poorly; Grade III/IV	0.560 (0.480–0.652)	<0.001	0.554 (0.430–0.713)	<0.001
**Subtype**				
HR+/HER2–	0.585 (0.535–0.639)	<0.001	0.864 (0.657–1.524)	0.101
HR+/HER2+	0.587 (0.443–0.778)	<0.001	0.876 (0.780–1.815)	0.389
HR–/HER2–	0.575 (0.456–0.726)	<0.001	0.534 (0.374–0.762)	0.001
HR–/HER2+	0.881 (0.668–1.772)	0.183	0.881 (0.720–1.833)	0.349
**Stage, AJCC 7th**				
I	0.382 (0.348–0.419)	<0.001	0.455 (0.358–0.577)	<0.001
II	0.298 (0.269–0.332)	<0.001	0.439 (0.360–0.537)	<0.001
**T**				
T1	0.623 (0.560–0.692)	<0.001	0.563 (0.434–0.730)	<0.001
T2	0.496 (0.439–0.561)	<0.001	0.535 (0.426–0.672)	<0.001
**N**				
N0	0.567 (0.523–0.614)	<0.001	0.521 (0.435–0.624)	<0.001
N1	0.489 (0.399–0.599)	0.003	0.425 (0.175–1.033)	0.059
**Breast surgery type**				
BCS	0.584 (0.532–0.642)	<0.001	0.570 (0.464–0.699)	<0.001
Mastectomy	0.537 (0.454–0.635)	<0.001	0.549 (0.385–0.782)	0.001
**Radiation**				
Yes	0.625 (0.529–0.739)	<0.001	0.606 (0.417–0.881)	0.009
No/unknown	0.555 (0.508–0.607)	<0.001	0.538 (0.443–0.653)	<0.001
**Chemotherapy**				
Yes	0.638 (0.401–1.010)	0.170	0.536 (0.448–1.642)	0.304
No/unknown	0.574 (0.530–0.622)	<0.001	0.542 (0.453–0.650)	<0.001

*Multivariate Cox regression model*.

*All HRs refer to Surgery vs .non-surgery in the subgroup analysis*.

*AIA, American Indian/Alaska Native; API, Asian or Pacific Islander; NST, no special type; ILC, invasive lobular carcinoma; BCS, Breast reserving mastectomy/Lumpectomy; CI, confidence interval; OS overall survival; BCSS, breast cancer specific survival*.

a *“others” includes “Central portion of breast,” “Breast includes Nipple” and “Overlapping lesion of breast such as 3, 6, 9, 12 o'clock” as recorded in the SEER database*.

b *“favorable” includes tubular, mucinous and papillary; “Others” means histological types other than above four types*.

### Risk Covariates Related to Survival in Patients With and Without Surgery

To determine the multiple factors associated with OS and BCSS in patients with and without SLNB, the univariate and multivariate Cox proportional hazards regression models were performed. Initially, 14 variables were involved in the univariate analysis (Supplementary Table 4), and seven variables were found to be significant risk factors for OS and BCSS in both patients with and without SLNB: age at diagnosis, marital status, grade, subtype, T stage, breast surgery type, and radiation. Additionally, in the non-SLNB group, race, tumor location, and chemotherapy were risk factors for OS, and N stage was a risk factor for BCSS. In the SLNB group, race and N stage were factors for OS and BCSS, and chemotherapy was a risk factor for BCSS.

Subsequently, risk factors with a *P*-value <0.05 in univariate Cox analysis were considered in the multivariate analysis (Table 3). Multivariate analysis identified that six variables (age at diagnosis, marital status, grade, breast cancer subtype, T stage, and radiation) remained significantly associated with OS and BCSS in both groups.

**Table 3:** Multivariable Cox models for metastasis breast cancer patients in surgery and non-surgery set.

**Variables**	**OS**				**BCSS**			
	**No SLNB**		**SLNB**		**No SLNB**		**SLNB**	
	**HR (95% CI)**	***P***	**HR (95% CI)**	***P***	**HR (95% CI)**	***P***	**HR (95% CI)**	***P***
**Age at diagnosis, y**		<0.001		<0.001		0.005		<0.001
70–74	1.000 [Reference]		1.000 [Reference]		1.000 [Reference]		1.000 [Reference]	
75–79	1.249 (0.932–1.673)	0.135	1.453 (1.314–1.608)	<0.001	1.806 (0.940–3.469)	0.076	1.308 (1.085–1.577)	0.005
80–84	1.473 (1.125–1.930)	0.005	2.274 (2.052–2.520)	<0.001	1.501 (0.802–2.811)	0.204	1.434 (1.159–1.774)	0.001
85+	2.184 (1.690–2.822)	<0.001	3.440 (3.072–3.852)	<0.001	2.102 (1.163–3.801)	0.014	1.946 (1.533–2.471)	<0.001
**Race**		0.003		<0.001				
White	1.000 [Reference]		1.000 [Reference]		–	–	–	–
Black	0.878 (0.690–1.116)	0.289	1.108 (0.971–1.265)	0.127	–	–	–	–
AIA	2.022 (0.901–4.536)	0.087	1.730 (1.073–2.791)	0.025	–	–	–	–
API	0.597 (0.431–0.827)	0.002	0.589 (0.490–0.709)	<0.001	–	–	–	–
**Marital**								
Unmarried	1.000 [Reference]		1.000 [Reference]		1.000 [Reference]		1.000 [Reference]	
Married	0.802 (0.693–0.927)	0.003	0.789 (0.729–0.853)	<0.001	0.557 (0.389–0.796)	0.001	0.846 (0.724–0.988)	0.035
**Grade**		0.003		<0.001		<0.001		<0.001
Well	1.000 [Reference]		1.000 [Reference]		1.000 [Reference]		1.000 [Reference]	
Moderately	1.053 (0.909–1.220)	0.489	1.106 (1.006–1.216)	0.036	1.145 (0.774–1.694)	0.497	1.342 (1.059–1.701)	0.015
Poorly	1.414 (1.170–1.709)	<0.001	1.434 (1.277–1.611)	<0.001	2.419 (1.566–3.736)	<0.001	2.730 (2.114–3.525)	<0.001
**Subtype**		0.014		0.038		0.019		0.030
HR+/HER2–	1.000 [Reference]		1.000 [Reference]		1.000 [Reference]		1.000 [Reference]	
HR+/HER2+	1.425 (1.122–1.808)	0.004	1.149 (0.992–1.331)	0.063	1.348 (0.801–2.270)	0.261	1.387 (1.055–1.825)	0.019
HR–/HER2–	1.531 (1.229–1.908)	<0.001	1.518 (1.344–1.715)	<0.001	2.836 (1.931–4.165)	<0.001	2.513 (2.040–3.096)	<0.001
HR–/HER2+	1.262 (0.828–1.923)	0.279	1.119 (0.899–1.393)	0.312	2.119 (1.112–4.037)	0.022	1.868 (1.319–2.644)	<0.001
**T**								
T1	1.000 [Reference]		1.000 [Reference]		1.000 [Reference]		1.000 [Reference]	
T2	2.026 (1.779–2.307)	<0.001	1.512 (1.393–1.642)	<0.001	2.732 (2.035–3.669)	<0.001	2.354 (2.007–2.760)	<0.001
**N**								
N0	–		1.000 [Reference]		1.000 [Reference]		1.000 [Reference]	
N1	–		1.451 (1.306–1.611)	<0.001	1.328 (0.533–3.311)	0.542	2.396 (2.008–2.858)	<0.001
**Breast surgery type**								
BCS	1.000 [Reference]		1.000 [Reference]		1.000 [Reference]		1.000 [Reference]	
Mastectomy	1.026 (0.867–1.214)	0.764	1.084 (0.989–1.189)	0.083	0.862 (0.600–1.239)	0.424	1.034 (0.865–1.235)	0.711
**Radiation**								
No/unknown	1.000 [Reference]		1.000 [Reference]		1.000 [Reference]		1.000 [Reference]	
Yes	0.592 (0.500–0.702)	<0.001	0.627 (0.573–0.686)	<0.001	0.548 (0.371–0.810)	0.003	0.589 (0.493–0.705)	<0.001
**Chemotherapy**								
No/unknown	1.000 [Reference]		–		–		1.000 [Reference]	
Yes	0.512 (0.317–0.826)	0.006	–		–		0.905 (0.736–1.114)	0.349

*AIA, American Indian/Alaska Native; API, Asian or Pacific Islander; NST, no special type; BCS, Breast reserving mastectomy/Lumpectomy; CI, confidence interval; SLNB, sentinel lymph node biopsy; OS overall survival; BCSS, breast cancer specific survival*.

### Construction of Nomograms and Validation

**Figure 3:**
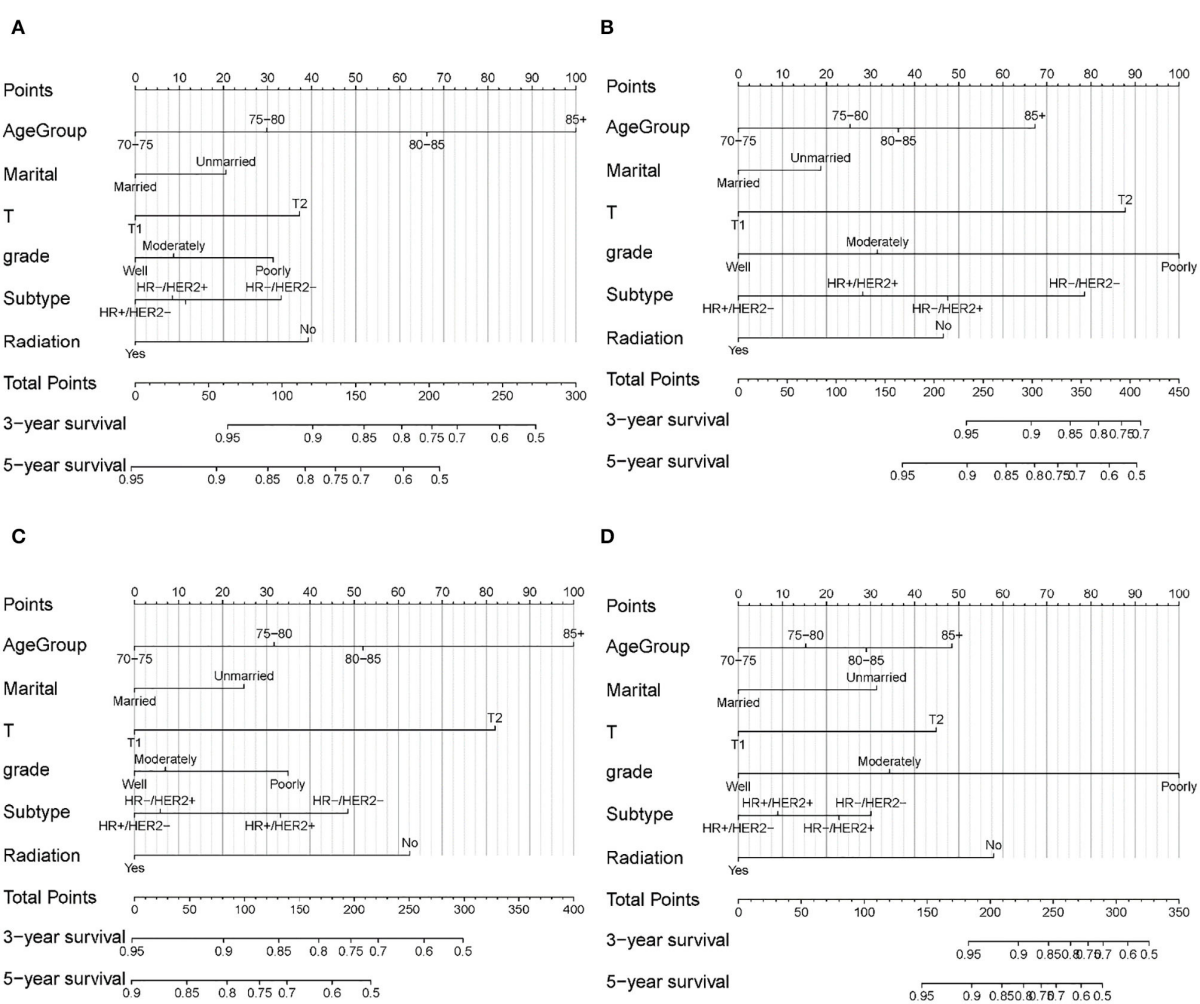
Nomogram for predicting 3- and 5-year OS and BCSS in elderly patients with early-stage breast cancer. **(A)** OS for patients with SLNB. **(B)** BCSS for patients with SLNB. **(C)** OS for patients without SLNB. **(D)** BCSS for patients without SLNB.

According to the results from multivariate Cox analysis (Table 3), six variables (age, marital status, T, grade, subtype, and radiation) were incorporated into nomograms to predict 3- and 5-year OS and BCSS for patients with and without SLNB (Figures 3A–D). According to the point scale in each nomogram, scores were signed for each variable (Table 4), and a total point can be calculated by adding all points based on patient's individual clinicopathological characteristics. A lower score was considered to have better prognosis. By comparing the survival outcomes predicted by the four separate nomograms, clinicians and patients can weigh the risk–benefit gained from SLNB and make a tailored decision.

**Table 4:** Scores of clinical variables in each nomogram.

**Variables**	**OS**		**BCSS**	
	**SLNB**	**No SLNB**	**SLNB**	**No SLNB**
**Age at diagnosis, y**				
70–74	0	0	0	0
75–79	30	32	25	15
80–84	66	52	36	29
85+	100	100	67	48
**Marital**				
Unmarried	21	25	19	31
Married	0	0	0	0
**Grade**				
Well	0	0	0	0
Moderately	9	7	32	34
Poorly	31	35	100	100
**Subtype**				
HR+/HER2–	0	0	0	0
HR+/HER2+	11	33	28	9
HR–/HER2–	33	49	79	30
HR–/HER2+	8	6	48	23
**T**				
T1	0	0	0	0
T2	37	82	88	45
**Radiation**				
No/unknown	39	63	47	58
Yes	0	0	0	0

**Figure 4:**
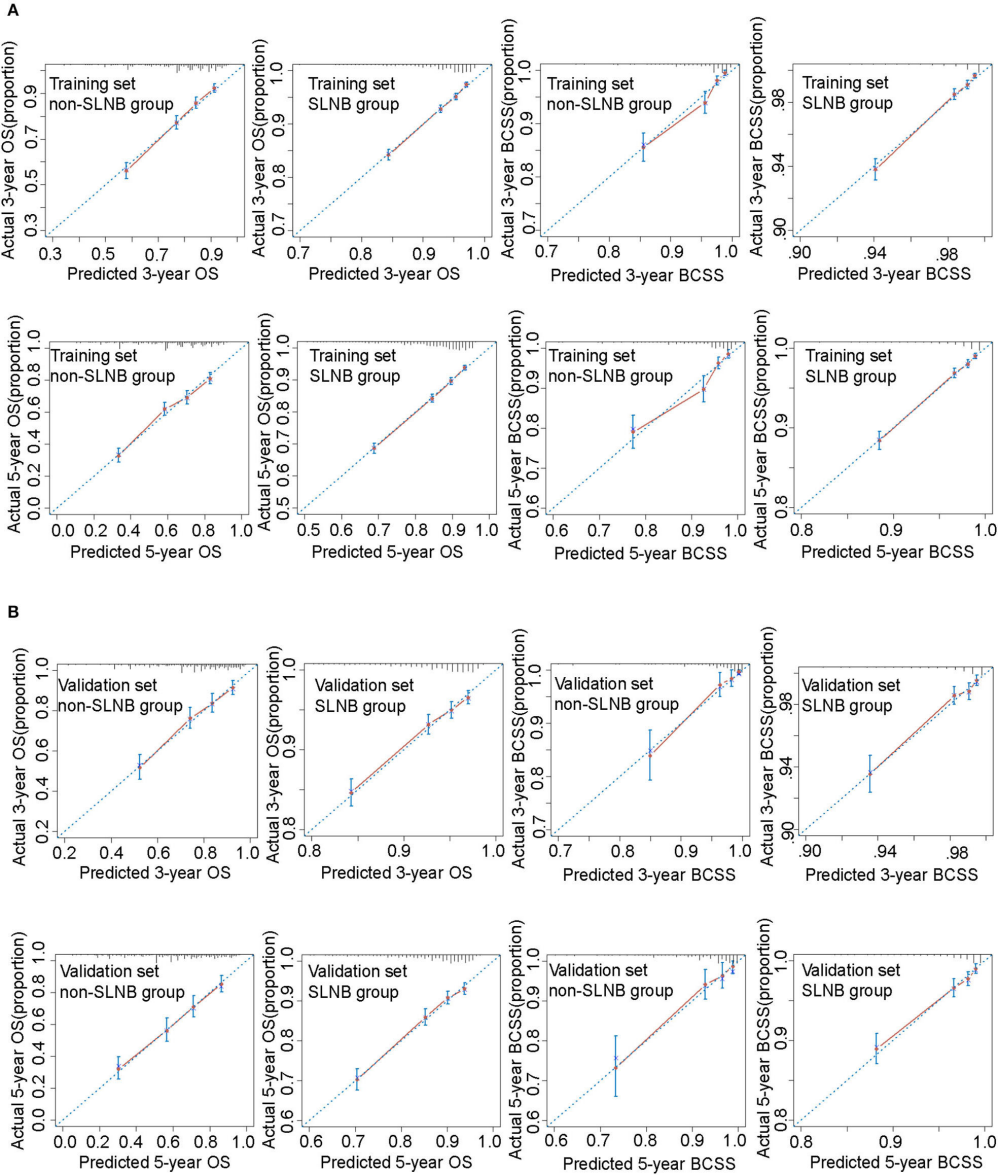
Calibration curves for these nomograms in training set **(A)** and validation set **(B)**. The 45° blue dotted line represents the ideal reference, which means the nomogram-predicted survival probabilities (*x*-axis) exactly match the actual survival proportions (*y*-axis). Red dots represent nomogram-predicted probabilities for each group, and blue error bars represent the 95% CIs of these estimates.

Nomograms were validated internally and externally using the training set and the validation set. The C-index of the four nomograms ranged from 0.695 to 0.785 in the internal validation and 0.687 to 0.820 in the external validation (Supplementary Table 5). Calibration curves for 3- and 5-year OS and BCSS prediction showed good coordination between predictions of the model and observed outcomes (Figure 4). Both the internal validation and the external validation demonstrated a sufficient accuracy of the models.

### Survival Benefit in Risk Stratification Group

**Figure 5:**
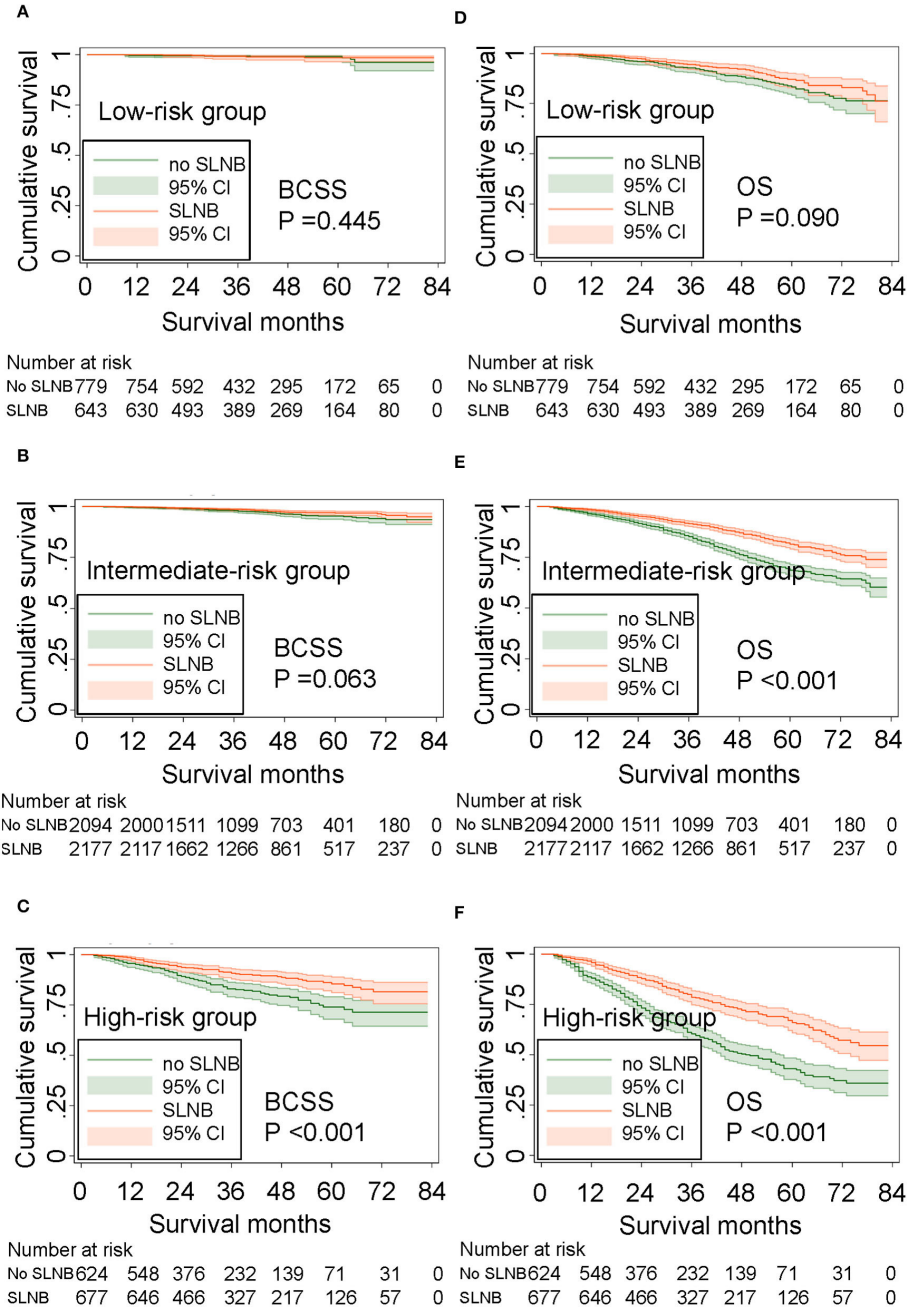
Survival benefit of SLNB in the risk stratification groups. **(A)** BCSS in the low-risk group, **(B)** BCSS in the intermediate-risk group, **(C)** BCSS in the high-risk group. **(D)** OS in the low-risk group, **(E)** OS in the intermediate-risk group, and **(F)** OS in the high-risk group.

For older patients, the risk factors associated with OS are complex, and BCSS is an important outcome during treatments for breast cancer patients. To further determine those who could benefit from SLNB and those who do not, we built a risk stratification based on the nomogram that predicted BCSS for patients undergoing SLNB, and we combined the SLNB cohort in the validation set (*n* = 8,665) and all non-SLNB cohort (*n* = 5,287) as a new validation set to assess the survival benefit of SLNB in each stratified risk group. In order to reduce the selection bias and balance baseline between the SLNB group and non-SLNB group, propensity score matching was performed using logistic regression with a caliper width of 0.01 and a 1:1 ratio without replacement. After matching for age, marital status, T stage, grade, subtype, and radiation, which were considered as independent prognostic factors, 3,497 pair patients were included. The distribution of propensity score for matched and unmatched patients is shown in Supplementary Figure 1. The score range in the risk stratification model was defined as low risk (total score <70), intermediate risk (total score 70–210), and high risk (total score >210). According to the risk stratification model, all matched patients were stratified into the low-risk group (1,422/6,994, 20.3%), intermediate-risk group (4,271/6,994, 61.1%), and high-risk group (1,301/6,994, 18.6%). Kaplan–Meier plots (Figures 5A–F) and log-rank test in the stratified risk groups showed that SLNB significantly improved OS [HR 0.485 (0.400–0.588), *P* < 0.001] and BCSS [HR 0.541 (0.395–0.742), *P* < 0.001] in the high-risk group, but not in the low-risk group [OS: HR 0.749 (0.537–1.046), *P* = 0.090; BCSS: HR 0.674 (0.245–1.856), *P* = 0.445]. In the intermediate-risk group, SLNB exerted a protective factor for OS [HR 0.559 (0.481–0.651), *P* < 0.001] but not for BCSS [HR 0.693 (0.470–1.020), *P* = 0.063].

## Discussion

Less is known about survival outcomes of sparing SLNB in elderly breast cancer patients. SLNB is associated with improved quality of life and with reduced complications compared with ALND and has become the golden process of axillary treatment of patients with early-stage breast cancer. However, the side effects of SLNB cannot be neglected in elderly patients. It is reported that prevalence of lymphedema 1 year after SLNB ranges between 3 and 17%, and for pain, prevalence rates between 3.3 and 56.6% have been reported in SLN-negative breast cancer patients (5, 20, 21). Another study conducted by Mandelblatt found that patients 67 years or older who underwent SLNB and/or ALND suffered from three times the incidence of arm complications than those who did not receive any axillary surgery (6). Is there a subset of elderly patients who can avoid SLNB without sacrificing survival? In the present study which included 39,962 ≥70-year-old patients diagnosed with primary early-stage breast cancer from 2010 to 2015 in the SEER program, the result of multivariate COX analysis indicated that SLNB was an independent prognostic factor for OS and BCSS. However, to our surprise, in our risk stratification model, we found that while SLNB could prolong OS and BCSS in the high-risk group, there is indeed a low-risk elderly population who did not gain survival benefit from SLNB, which means that the low-risk population could avoid the morbidities of SLNB at no expense of survival and that it will be more cost-effective for elderly patients. We believe these nomograms and the risk stratification model can be useful for clinicians and patients to fully consider the risk–benefit balance of SLNB.

Given the potential complications of SLNB for the elderly, researchers have been trying to explore the possibility of exemption from SLNB. Published studies addressing this question have yielded somewhat inconsistent conclusions. Earlier studies suggested that SLNB should be recommended to elderly breast cancer because the presence of axillary metastasis may influence subsequent adjuvant therapy decisions (9, 10, 12). However, a recent study with a cohort of 492 patients conducted by Blackhall et al. (13) showed that elderly patients with positive nodes were significantly less likely to receive chemotherapy than younger patients, which suggested that nowadays, postoperative treatment strategies depend more on the tumor's biological characteristics than on the result of axillary staging alone. Several studies have reported that SLNB could be spared in selected elderly patients due to the very low incidence of axillary disease in this age population (8, 22–24). A retrospective study reported that incomplete axillary staging did not exert a worse regional control and 10-year OS in 75 years or older patients with two or more comorbidities (8). A meta-analysis including 692 patients of two randomized controlled trials (RCTs) found that axillary staging in elderly patients with negative nodes could reduce regional recurrence (RR 0.24, 95% CI: 0.06–0.95, *P* = 0.04) but did not impact OS or BCSS (23). A recent research using a propensity score matching cohort based on the SEER found that 50- to 79-year-old patients who had a grade I tumor with size <1 cm and favorable histology did not gain a better BCSS from axillary staging (24). However, the individual effects of SLNB remained unknown because the axillary staging in those studies included SLNB and ALND. Notwithstanding the fact that another retrospective study involving a total of only 141 patients pointed out that the probability of omitting SLNB increased with age in 70 years or older patients with ER-positive, HER2-negative, and clinical T1N0 invasive breast cancer (14), few studies have accurately pointed out a specific population that would be exempt from SLNB. In the present study, patients aged 70 years or more were defined as elderly, which is in keeping with the general literature, where 70 years has become an accepted definition (10–13, 22). Deaths due to breast cancer (23.8%, 941/3,952) are low in relation with elderly patients, which may be explained by low median follow-up time (39 months), as well as the fact that older people are more likely to die from multiple comorbidities such as cardiovascular disease, diabetes mellitus, and other chronic diseases compared with younger people. We established a risk stratification model based on the score of the nomogram which predicted the BCSS of patients undergoing SLNB with a good validation. The characteristics of the low-risk group, which gets scores <70, included a relatively younger age, T1 stage, good grade, positive hormone receptor, and radiation. As reported in the Results section, after adjusting for potential confounding factors, SLNB still exerted a protective factor for the entire training cohort. The proportion of the low-risk group, though lower, is still 20.3%. It makes sense that they could be relatively safe to spare SLNB due to equivalent survival outcomes. With regard to the high-risk group (score >210), patients often had a larger tumor, worse grade, and triple negative breast cancer without radiation, and in those cases, SLNB cannot be omitted for fear of undertreatment due to decreased axillary staging. As for the intermediate-risk group, SLNB did not improve the BCSS, but the OS was extended due to some potential unknowns, and the decision to have SLNB or not should be made with caution in these patients and should take full consideration of patient's informed consent. More than half of the patients were classified into the intermediate-risk group (61.1%), and robust evidence from prospective trials is urgently needed.

In the absence of evidence demonstrating survival outcomes of omitting SLNB, several ongoing prospective randomized clinical trials are evaluating whether SLNB can be avoided in early breast cancer patients treated with BCS, whole-breast irradiation, and standard adjuvant surgery. The SOUND trial, a non-inferiority trial, is recruiting breast cancer patients with a ≤ 2 cm tumor and a negative axilla assessed by axillary ultrasound. Eligible patients will be randomized into an SLNB group and no-axillary-surgery group. The primary endpoint is distant disease-free survival (DFS), and secondary endpoints are the cumulative incidence of distant or axillary recurrences, DFS and OS (15). In the INSEMA trial, patients who have a <5 cm tumor, estimated by preoperative imaging techniques, will be randomized to the no-axillary-surgery group and SLNB group. A negative core biopsy or fine needle aspiration biopsy of the sonographically suspected lymph node is required before randomization. The DFS will be assessed at 5 years (25). The inclusion criteria of the Dutch BOOG 2013-08, with endpoints of regional recurrences, DFS, and OS at 5 and 10 years, are somehow similar to those in the INSEMA (26). The results of these well-designed clinical trials were eagerly awaited. Heretofore, few guidelines have explicitly stated the application of SLNB in the elderly. The version 3.2020 NCCN states that “the performance of axillary staging may be considered optional” for elderly patients or those with serious comorbidities (27), and the North American guidelines suggest that women >70 years old diagnosed with cN0 invasive breast cancer with positive hormone receptor should avoid routine use of SLNB (28). However, current UK and Scottish guidelines state that if fit for surgery, this population should be offered SLNB (29, 30). The acceptable balance between risk and benefit varies among clinicians and patients; therefore, it is hard to set a specific threshold to determine whether SLNB should be applied or not. Not only did our study provide nomograms to accurately predict 3- and 5-year OS and BCSS in elderly patients with T1–T2 breast cancer, but the risk stratification model also makes it easy to quickly determine whether one will benefit from SLNB. For example, a 78-year-old married woman was diagnosed clinically with T1N0 breast cancer. The result of her biopsy reported that the tumor histology was invasive breast cancer with good differentiation and that the breast subtype was HR–/HER2+. She plans to receive BCS, postoperative radiation, and targeted therapy. By comparing the prediction of OS and BCSS using our nomogram (Supplementary Table 6), whether this patient underwent SLNB or not, the predictions of 3- and 5-year BCSS were over 95%. If she underwent SLNB, the predictive 3- and 5-year OS rates were >95 and >90%, respectively. If SLNB was omitted, the predictive 3- and 5-year OS would be >90 and 87%, respectively. Given that she got a score of 38 in the nomogram, which predicts BCSS for patients undergoing SLNB, she was categorized into the low-risk group, suggesting that there was no significant difference in the OS and BCSS she received from SLNB and no SLNB. Therefore, clinicians and patients can individually determine the risk–benefit of SLNB and make the appropriate decision to proceed or spare SLNB based on an individualized threshold and patient's preferences.

To our knowledge, this study is the first to establish nomograms based on a large SEER data to evaluate whether SLNB can be omitted in elderly patients. Notwithstanding several strengths of this study, including a large population, strict stratification analysis for nomogram construction, and sufficient internal and external validation, the nomograms must be interpreted with caution due to several limitations. Firstly, since some detailed information was not reported in SEER, there may be some potential confounding factors that we did not take into consideration, such as multigene signature assessment, endocrine therapy, targeted therapy, and comorbidities (cardiovascular disease, diabetes mellitus, and other chronic diseases). As a result, the benefit of omitting SLNB in elderly patients may be underestimated because they are often faced with significant comorbidities and cannot tolerate aggressive complications of surgery, but standard adjuvant systemic therapy could compensate for the risk of no SLNB. The second was the inherent limitation of a retrospective study based on the SEER dataset. Since no variable in the current SEER database clearly specifies the type of lymph node surgery, we categorized removing one to five lymph nodes as SLNB according to the AJCC definition as other researchers did (31). This may lead to poorer prognosis in the SLNB group due to potentially insufficient SLNB with only five or fewer nodes removed. Nevertheless, it did not influence the conclusions in our study because survival analysis showed that the OS of the SLNB group was significantly better than that of the non-SLNB group, as well as BCSS. Additionally, although axillary recurrence rate is very low in all studies, some patients, even in the low-risk group, probably had axillary recurrences which need ALND and adjuvant therapies. This is not known in the SEER database. The median follow-up was low (39 months) in the present study. Perhaps, for very elderly patients with comorbidities, 3-year survival results could be contributive for these patients with short life probability, independent of breast cancer. The most convincing evidence must come from comprehensively designed prospective randomized trials with more prognostic factors. We look forward to the results of the SOUND, the INSEMA, and other clinical trials which evaluate the effect of omitting SLNB on long-term outcomes, particularly in elderly patients.

## Conclusion

Well-validated nomograms and a risk stratification model were constructed to evaluate survival benefit from SLNB in elderly patients with early-stage breast cancer. SLNB was important for patients in the high-risk group but could be omitted in the low-risk group without sacrificing survival. This study could assist clinicians and elderly patients to weigh the risk–benefit of SLNB and make individualized decisions. We look forward to more powerful evidence from prospective trials.

## Data Availability Statement

The datasets presented in this study can be found in online repositories. The names of the repository/repositories and accession number(s) can be found below: https://seer.cancer.gov/.

## Ethics Statement

This study was reviewed and approved by the institutional review board of West China Hospital.

## Author Contributions

ZD and QL contributed to conception and design of the study. LX, NW, JQ, QT, JY, and TH organized the database and performed the statistical analysis. LX and JQ prepared the figures and tables. LX, NW and ZD contributed to the manuscript writing and revision. All authors contributed to the article and approved the submitted version.

## Conflict of Interest

The authors declare that the research was conducted in the absence of any commercial or financial relationships that could be construed as a potential conflict of interest.
