# Supplementary material for: Predicting Survival Benefit of Sparing Sentinel Lymph Node Biopsy in Low-Risk Elderly Patients With Early Breast Cancer: A Population-Based Analysis
Source: Front Oncol. 2020 Sep 11;10:1718. doi: 10.3389/fonc.2020.01718 (PMC7517716; doi:10.3389/fonc.2020.01718)

Supplementary Material

**1 Supplementary Figures and Tables**

- 1. **Supplementary Tables**

**Supplementary Table 1** Demographic and disease characteristics of the study population

**Supplementary Table 2** Univariate Cox models for breast cancer patients in training set

**Supplementary Table 3** multivariate Cox models for breast cancer patients in training set

**Supplementary Table 4** Univariate Cox models for patients in surgery and non-surgery cohort

**Supplementary Table 5** C-index for internal and external validation

**Supplementary Table 6** The survival outcomes predicted by separate nomograms for the case used as an example

- 1. **Supplementary Figures**

**Supplementary Figure1** The distribution of propensity score for matched and unmatched patients

**1.1 Supplementary Tables**

**Supplementary Table 1**. Demographic and disease characteristics of the study population

| **Variables** | | All patients |  | Training cohort |  | Validation cohort | *P*‐value ^c^ |
| --- | --- | --- | --- | --- | --- | --- | --- |
|  |  | n(%) (n=39962) |  | n(%) (n=29971) |  | n(%) (n=9991) |  |
| **Age at diagnosis, y** | | |  |  |  |  | 0.576 |
|  | **70-74** | 16146 (40.4) |  | 12117 (40.4) |  | 4029 (40.3) |  |
|  | **75-79** | 11336 (28.4) |  | 8485 (28.3) |  | 2851 (28.5) |  |
|  | **80-84** | 7398 (18.5) |  | 5522 (18.4) |  | 1876 (18.8) |  |
|  | **85+** | 5082 (12.7) |  | 3847 (12.8) |  | 1235 (12.4) |  |
| **Race** | |  |  |  |  |  | 0.307 |
|  | **White** | 34189 (85.6) |  | 25590 (85.4) |  | 8599 (86.1) |  |
|  | **Black** | 2978 (7.5) |  | 2252 (7.5) |  | 726 (7.3) |  |
|  | **AIA** | 164 (0.4) |  | 121 (0.4) |  | 43 (0.4) |  |
|  | **API** | 2631 (6.6) |  | 2008 (6.7) |  | 623 (6.2) |  |
| **Marital** | |  |  |  |  |  | 0.686 |
|  | **Unmarried** | 21339 (53.4) |  | 15986 (53.3) |  | 5353 (53.6) |  |
|  | **Married** | 18623 (46.6) |  | 13985 (46.7) |  | 4638 (46.4) |  |
| **Benign or borderline tumors history** | | |  |  |  |  | 0.601 |
|  | **No** | 39687 (99.3) |  | 29769 (99.3) |  | 9918 (99.3) |  |
|  | **Yes** | 275 (0.7) |  | 202 (0.7) |  | 73 (0.7) |  |
| **Lateral** | |  |  |  |  |  | 0.669 |
|  | **Right** | 19375 (48.5) |  | 14512 (48.4) |  | 4863 (48.7) |  |
|  | **Left** | 20587 (51.5) |  | 15459 (51.6) |  | 5128 (51.3) |  |
| **Tumor location** | |  |  |  |  |  | 0.404 |
|  | **Outer quadrant and axillary tail** | 16677 (41.7) |  | 12547 (41.9) |  | 4130 (41.3) |  |
|  | **Inner quadrant** | 8391 (21.0) |  | 6310 (21.1) |  | 2081 (20.8) |  |
|  | **Others ^a^** | 14894 (37.3) |  | 11114 (37.1) |  | 3780 (37.8) |  |
| **Histology (ICD-O-3) ^b^** | | |  |  |  |  | 0.236 |
|  | **NST** | 33167 (83.0) |  | 24858 (82.9) |  | 8309 (83.2) |  |
|  | **ILC** | 4111 (10.3) |  | 3089 (10.3) |  | 1022 (10.2) |  |
|  | **Favorable** | 1863 (4.7) |  | 1400 (4.7) |  | 463 (4.6) |  |
|  | **Metaplastic** | 212 (0.5) |  | 149 (0.5) |  | 63 (0.6) |  |
|  | **others** | 609 (1.5) |  | 475 (1.6) |  | 134 (1.3) |  |
| **Grade** | |  |  |  |  |  | 0.572 |
|  | **Well, I** | 12788 (32.0) |  | 9560 (31.9) |  | 3228 (32.3) |  |
|  | **Moderately, II** | 19046 (47.7) |  | 14282 (47.7) |  | 4764 (47.7) |  |
|  | **Poorly, III/IV** | 8128 (20.3) |  | 6129 (20.4) |  | 1999 (20.0) |  |
| **Subtype** | |  |  |  |  |  | 0.207 |
|  | **HR+/HER2-** | 33312 (83.4) |  | 24995 (83.4) |  | 8317 (83.2) |  |
|  | **HR+/HER2+** | 2559 (6.4) |  | 1879 (6.3) |  | 680 (6.8) |  |
|  | **HR-/HER2-** | 3191 (8.0) |  | 2419 (8.1) |  | 772 (7.7) |  |
|  | **HR-/HER2+** | 900 (2.3) |  | 678 (2.3) |  | 222 (2.2) |  |
| **ER** | |  |  |  |  |  | 0.497 |
|  | **Negative** | 4351 (10.9) |  | 3282 (11.0) |  | 1069 (10.7) |  |
|  | **Positive** | 35611 (89.1) |  | 26689 (89.0) |  | 8922 (89.3) |  |
| **PR** | |  |  |  |  |  | 0.824 |
|  | **Negative** | 8489 (21.2) |  | 6375 (21.3) |  | 2114 (21.2) |  |
|  | **Positive** | 31473 (78.8) |  | 23596 (78.7) |  | 7877 (78.8) |  |
| **HER2** | |  |  |  |  |  | 0.132 |
|  | **Negative** | 36503 (91.3) |  | 27414 (91.5) |  | 9089 (91.0) |  |
|  | **Positive** | 3459 (8.7) |  | 2557 (8.5) |  | 902 (9.0) |  |
| **Stage, AJCC 7^th^** | | |  |  |  |  | 0.998 |
|  | **I** | 28745 (71.9) |  | 21559 (71.9) |  | 7186 (71.9) |  |
|  | **II** | 11217 (28.1) |  | 8412 (28.1) |  | 2805 (28.1) |  |
| **T** | |  |  |  |  |  | 0.912 |
|  | **T1** | 30060 (75.2) |  | 22540 (75.2) |  | 7520 (75.3) |  |
|  | **T2** | 9902 (24.8) |  | 7431 (24.8) |  | 2471 (24.7) |  |
| **N** | |  |  |  |  |  | 0.026 |
|  | **N0** | 36072 (90.3) |  | 27111 (90.5) |  | 8961 (89.7) |  |
|  | **N1** | 3890 (9.7) |  | 2860 (9.5) |  | 1030 (10.3) |  |
| **Axillary surgery** | |  |  |  |  |  | 0.578 |
|  | **No SLNB** | 5287 (13.2) |  | 3982 (13.3) |  | 1305 (13.1) |  |
|  | **SLNB** | 34675 (86.8) |  | 26089 (86.7) |  | 8686 (86.9) |  |
| **Breast surgery type** | | |  |  |  |  | 0.239 |
|  | **BCS** | 30601 (76.6) |  | 22994 (76.7) |  | 7607 (76.1) |  |
|  | **Mastectomy** | 9361 (23.4) |  | 6977 (23.3) |  | 2384 (23.9) |  |
| **Radiation** | |  |  |  |  |  | 0.298 |
|  | **No/unknown** | 19737 (49.4) |  | 14848 (49.5) |  | 4889 (48.9) |  |
|  | **Yes** | 20225 (50.6) |  | 15123 (50.5) |  | 5102 (51.1) |  |
| **Chemotherapy** | |  |  |  |  |  | 0.903 |
|  | **No/unknown** | 35563 (89.0) |  | 26668 (89.0) |  | 8895 (89.0) |  |
|  | **Yes** | 4399 (11.0) |  | 3303 (11.0) |  | 1096 (11.0) |  |
| **Follow-up months, mean(SD)** | | 41.33 (20.70) |  | 41.37 (20.70) |  | 41.21 (20.71) | 0.494 |
| Note: AIA American Indian/Alaska Native; API Asian or Pacific Islander; NST no special type; ILC invasive lobular carcinoma; SLNB sentinel lymph node biopsy; BCS Breast reserving mastectomy/Lumpectomy   1. “others” includes “Central portion of breast”, “Breast includes Nipple” and “Overlapping lesion of breast such as 3, 6, 9, 12 o’clock” as recorded in the SEER database 2. “favorable” includes tubular, mucinous and papillary; “Others” means histological types other than above four types. 3. The comparison results between training set and validation set using chi‐squared test. | | | | | | | |

**Supplementary Table 2** Univariate Cox models for breast cancer patients in training set

| **Variables** | | **OS** | |  | **BCSS** | |
| --- | --- | --- | --- | --- | --- | --- |
|  |  | **HR (95% CI)** | ***P*-value** |  | **HR (95% CI)** | ***P*-value** |
| **Age at diagnosis, y** | |  | <0.001 |  |  | <0.001 |
|  | **70-74** | 1.000 [Reference] |  |  | 1.000 [Reference] |  |
|  | **75-79** | 1.549 (1.409-1.703) | <0.001 |  | 1.445 (1.212-1.724) | <0.001 |
|  | **80-84** | 2.681 (2.445-2.940) | <0.001 |  | 1.794 (1.489-2.161) | <0.001 |
|  | **85+** | 5.396 (4.939-5.894) | <0.001 |  | 3.527 (2.954-4.211) | <0.001 |
| **Race** | |  | <0.001 |  |  | 0.434 |
|  | **White** | 1.000 [Reference] |  |  | 1.000 [Reference] |  |
|  | **Black** | 1.120 (0.999-.256) | 0.052 |  | 1.424 (1.151-1.762) | 0.001 |
|  | **AIA** | 1,532 (1.016-2.308) | 0.042 |  | 1.993 (0.946-4.193) | 0.069 |
|  | **API** | 0.595 (0.507-0.699) | <0.001 |  | 0.727 (0.536-0.984) | 0.039 |
| **Marital** | |  |  |  |  |  |
|  | **Unmarried** | 1.000 [Reference] |  |  | 1.000 [Reference] |  |
|  | **Married** | 0.657 (0.584-0.740) | <0.001 |  | 0.714 (0.559-0.914) | <0.001 |
| **Benign or borderline tumors history** | | |  |  |  |  |
|  | **No** | 1.000 [Reference] |  |  | 1.000 [Reference] |  |
|  | **Yes** | 1.112 (0.785-1.575) | 0.549 |  | 1.326 (0.688-2.556) | 0.400 |
| **Lateral** | |  |  |  |  |  |
|  | **Right** | 1.000 [Reference] |  |  | 1.000 [Reference] |  |
|  | **Left** | 0.994 (0.934-1.058) | 0.854 |  | 0.993 (0.874-1.129) | 0.919 |
| **Tumor location** | |  | 0.004 |  |  | 0.141 |
|  | **Outer quadrant** | 1.000 [Reference] |  |  | 1.000 [Reference] |  |
|  | **Inner quadrant** | 0.900 (0.825-0.981) | 0.017 |  | 0.892 (0.746-1.067) | 0.211 |
|  | **Others ^a^** | 1.111 (1.036-1.190) | 0.003 |  | 1.115 (0.967-1.285) | 0.131 |
| **Histology (ICD-O-3) ^b^** | |  | 0.016 |  |  | 0.824 |
|  | **NST** | 1.000 [Reference] |  |  | 1.000 [Reference] |  |
|  | **ILC** | 0.977 (0.880-1.085) | 0.666 |  | 0.846 (0.674-1.061) | 0.148 |
|  | **Favorable** | 1.036 (0.896-1.197) | 0.635 |  | 0.457 (0.297-0.705) | <0.001 |
|  | **metaplastic** | 2.251 (1.648-3.075) | 0.000 |  | 5.215 (3.445-7.894) | <0.001 |
|  | **others** | 1.185 (0.942-1.489) | 0.147 |  | 0.972 (0.583-1.620) | 0.912 |
| **Grade** | |  | <0.001 |  |  | <0.001 |
|  | **Well; Grade I** | 1.000 [Reference] |  |  | 1.000 [Reference] |  |
|  | **Moderately; Grade II** | 1.209 (1.119-1.306) | <0.001 |  | 1.678 (1.378-2.044) | <0.001 |
|  | **Poorly; Grade III/IV** | 1.911 (1.756-2.079) | <0.001 |  | 5.51 (4.560-6.662) | <0.001 |
| **ER** | |  |  |  |  |  |
|  | **Negative** | 1.000 [Reference] |  |  | 1.000 [Reference] |  |
|  | **Positive** | 0.581 (0.535-0.632) | <0.001 |  | 0.251 (0.218-0.288) | <0.001 |
| **PR** | |  |  |  |  |  |
|  | **Negative** | 1.000 [Reference] |  |  | 1.000 [Reference] |  |
|  | **Positive** | 0.654 (0.611-0.701) | <0.001 |  | 0.316 (0.279-0.360) | <0.001 |
| **HER2** | |  |  |  |  |  |
|  | **Negative** | 1.000 [Reference] |  |  | 1.000 [Reference] |  |
|  | **Positive** | 1.314 (1.185-1.457) | <0.001 |  | 1.934 (1.612-2.321) | <0.001 |
| **T** | |  |  |  |  |  |
|  | **T1** | 1.000 [Reference] |  |  | 1.000 [Reference] |  |
|  | **T2** | 2.200 (2.063-2.345) | <0.001 |  | 3.979 (3.500-4.524) | <0.001 |
| **N** | |  |  |  |  |  |
|  | **N0** | 1.000 [Reference] |  |  | 1.000 [Reference] |  |
|  | **N1** | 1.367 (1.239-1.509) | <0.001 |  | 2.539 (2.160-2.984) | <0.001 |
| **Axillary surgery** | |  |  |  |  |  |
|  | **No SLNB** | 1.000 [Reference] |  |  | 1.000 [Reference] |  |
|  | **SLNB** | 0.346 (0.323-0.371) | <0.001 |  | 0.455 (0.391-0.530) | <0.001 |
| **Breast surgery type** | |  |  |  |  |  |
|  | **BCS** | 1.000 [Reference] |  |  | 1.000 [Reference] |  |
|  | **Mastectomy** | 1.474 (1.377—1.577) | <0.001 |  | 1.794 (1.569-2.050) | <0.001 |
| **Radiation** | |  |  |  |  |  |
|  | **No/unknown** | 1.000 [Reference] |  |  | 1.000 [Reference] |  |
|  | **Yes** | 0.429 (0.402-0.459) | <0.001 |  | 0.441 (0.384-0.505) | <0.001 |
| **Chemotherapy** | |  |  |  |  |  |
|  | **No/unknown** | 1.000 [Reference] |  |  | 1.000 [Reference] |  |
|  | **Yes** | 0.779 (0.696-0.872) | <0.001 |  | 1.863 (1.578-2.200) | <0.001 |
| Note: AIA American Indian/Alaska Native; API Asian or Pacific Islander; NST no special type; ILC invasive lobular carcinoma; SLNB sentinel lymph node biopsy; BCS Breast reserving mastectomy/Lumpectomy; OS overall survival; BCSS, breast cancer specific survival. | | | | | | |

**Supplementary Table 3** multivariate Cox models for breast cancer patients in training set

| **Variables** | | **OS** | |  | **BCSS** | |
| --- | --- | --- | --- | --- | --- | --- |
|  |  | **HR (95% CI)** | ***P*-value** |  | **HR (95% CI)** | ***P*-value** |
| **Age at diagnosis, y** | |  | <0.001 |  |  | <0.001 |
|  | **70-74** | 1.000 [Reference] |  |  | 1.000 [Reference] |  |
|  | **75-79** | 1.410 (1.281-1.551) | <0.001 |  | 1.319 (1.103-1.577) | 0.002 |
|  | **80-84** | 2.056 (1.866-2.265) | <0.001 |  | 1.363 (1.119-1.661) | 0.002 |
|  | **85+** | 3.018 (2.725-3.343) | <0.001 |  | 1.842 (1.496-2.268) | <0.001 |
| **Race** | |  | 0.069 |  |  |  |
|  | **White** | 1.000 [Reference] |  |  | - |  |
|  | **Black** | 1.041 (0.929-1.172) | 0.473 |  | - | - |
|  | **AIA** | 1.827 (1.211-2.756) | 0.004 |  | - | - |
|  | **API** | 0.594 (0.506-0.698) | <0.001 |  | - | - |
| **Marital** | |  |  |  |  |  |
|  | **Unmarried** | 1.000 [Reference] |  |  | 1.000 [Reference] |  |
|  | **Married** | 0.797 (0.707-0.899) | <0.001 |  | 0.870 (0.680-1.114) | 0.08 |
| **Tumor location** | |  | 0.650 |  |  |  |
|  | **Outer quadrant and axillary tail** | 1.000 [Reference] |  |  | - |  |
|  | **Inner quadrant** | 0.913 (0.837-0.996) | 0.041 |  | - | - |
|  | **Others ^a^** | 1.020 (0.950-1.093) | 0.595 |  | - | - |
| **Histology (ICD-O-3) ^b^** | |  | 0.756 |  |  |  |
|  | **NST** | 1.000 [Reference] |  |  | - | - |
|  | **ILC** | 0.982 (0.882-1.094) | 0.745 |  | - | - |
|  | **Favorable** | 1.048 (0.903-1.216) | 0.538 |  | - | - |
|  | **metaplastic** | 1.227 (0.892-1.687) | 0.209 |  | - | - |
|  | **others** | 0.931 (0.739-1.172) | 0.542 |  | - | - |
| **Grade** | |  | <0.001 |  |  | <0.001 |
|  | **Well** | 1.000 [Reference] |  |  | 1.000 [Reference] |  |
|  | **Moderately** | 1.095 (1.011-1.186) | 0.025 |  | 1.295 (1.060-1.582) | 0.012 |
|  | **Poorly/undifferentiated** | 1.412 (1.278-1.561) | <0.001 |  | 2.489 (2.001-3.095) | <0.001 |
| **ER** | |  |  |  |  |  |
|  | **Negative** | 1.000 [Reference] |  |  | 1.000 [Reference] |  |
|  | **Positive** | 0.591 (0.435-0.800) | 0.001 |  | 0.354 (0.223-0.560) | <0.001 |
| **PR** | |  |  |  |  |  |
|  | **Negative** | 1.000 [Reference] |  |  | 1.000 [Reference] |  |
|  | **Positive** | 0.823 (0.748-0.906) | <0.001 |  | 0.592 (0.490-0.715) | <0.001 |
| **HER2** | |  |  |  |  |  |
|  | **Negative** | 1.000 [Reference] |  |  | 1.000 [Reference] |  |
|  | **Positive** | 1.314 (1.185-1.457) | 0.009 |  | 1.372 (1.014-2.146) | 0.040 |
| **T** | |  |  |  |  |  |
|  | **T1** | 1.000 [Reference] |  |  | 1.000 [Reference] |  |
|  | **T2** | 1.715 (1.378-2.135) | <0.001 |  | 3.979 (3.500-4.524) | 0.002 |
| **N** | |  |  |  |  |  |
|  | **N0** | 1.000 [Reference] |  |  | 1.000 [Reference] |  |
|  | **N1** | 1.471 (1.307-1.656) | <0.001 |  | 2.139 (1.761-2.598) | <0.001 |
| **Axillary surgery** | |  |  |  |  |  |
|  | **No SLNB** | 1.000 [Reference] |  |  | 1.000 [Reference] |  |
|  | **SLNB** | 0.568 (0.523-0.616) | <0.001 |  | 0.554 (0.464-0.662) | <0.001 |
| **Breast surgery type** | |  |  |  |  |  |
|  | **BCS** | 1.000 [Reference] |  |  | 1.000 [Reference] |  |
|  | **Mastectomy** | 1.051 (0.970-1.138) | 0.228 |  | 0.987 (0.844-1.155) | 0.874 |
| **Radiation** | |  |  |  |  |  |
|  | **No/unknown** | 1.000 [Reference] |  |  | 1.000 [Reference] |  |
|  | **Yes** | 0.618 (0.572-0.669) | <0.001 |  | 0.584 (0.499-0.688) | <0.001 |
| **Chemotherapy** | |  |  |  |  |  |
|  | **No/unknown** | 1.000 [Reference] |  |  | 1.000 [Reference] |  |
|  | **Yes** | 0.713 (0.628-0.811) | <0.001 |  | 0.827 (0.679-1.007) | 0.058 |
| Note: AIA American Indian/Alaska Native; API Asian or Pacific Islander; NST no special type; ILC invasive lobular carcinoma; SLNB sentinel lymph node biopsy; BCS Breast reserving mastectomy/Lumpectomy; OS overall survival; BCSS, breast cancer specific survival. | | | | | | |

**Supplementary Table 4.** Univariate Cox models for patients in surgery and non-surgery cohort

| **Variables** | | OS | | | | |  | BCSS | | | | |
| --- | --- | --- | --- | --- | --- | --- | --- | --- | --- | --- | --- | --- |
|  |  | No SLNB | |  | SLNB | |  | No SLNB | |  | SLNB | |
|  |  | HR (95% CI) | *P* |  | HR (95% CI) | *P* |  | HR (95% CI) | *P* |  | HR (95% CI) | *P* |
| **Age at diagnosis, y** | | | <0.001 |  |  | <0.001 |  |  | <0.001 |  |  | <0.001 |
|  | **70-74** | 1.000 [Reference] |  |  | 1.000 [Reference] |  |  | 1.000 [Reference] |  |  | 1.000 [Reference] |  |
|  | **75-79** | 1.379 (1.032-1.842) | 0.030 |  | 1.511 (1.367-1.671) | <0.001 |  | 1.872 (0.980-3.578) | 0.058 |  | 1.385 (1.151-1.665) | 0.001 |
|  | **80-84** | 1.802 (1.386-2.344) | <0.001 |  | 2.565 (2.319-2.838) | <0.001 |  | 1.849 (0.999-3.433) | 0.050 |  | 1.693 (1.382-2.075) | <0.001 |
|  | **85+** | 3.256 (2.552-4.155) | <0.001 |  | 4.560 (4.093-5.080) | <0.001 |  | 3.435 (1.940-6.084) | <0.001 |  | 2.964 (2.379-3.693) | <0.001 |
| **Race** | |  | 0.005 |  |  | <0.001 |  |  | 0.743 |  |  | 0.568 |
|  | **White** | 1.000 [Reference] |  |  | 1.000 [Reference] |  |  | 1.000 [Reference] |  |  | 1.000 [Reference] |  |
|  | **Black** | 0.948 (0.749-1.199) | 0.655 |  | 1.194 (1.047-1.361) | <0.001 |  | 1.049 (0.630-1.749) | 0.854 |  | 1.544 (1.221-1.952) | <0.001 |
|  | **AIA** | 1.634 (0.732-3.645) | 0.231 |  | 1.557 (0.967-2.509) | 0.069 |  | 2.881 (0.715-9.605) | 0.137 |  | 1.829 (0.759-4.410) | 0.179 |
|  | **API** | 0.600 (0.434-0.830) | <0.001 |  | 0.614 (0.510-0.738) | <0.001 |  | 0.812 (0.430-1.534) | 0.521 |  | 0.720 (0.510-1.017) | 0.062 |
| **Marital** | |  |  |  |  |  |  |  |  |  |  |  |
|  | **Unmarried** | 1.000 [Reference] |  |  | 1.000 [Reference] |  |  | 1.000 [Reference] |  |  | 1.000 [Reference] |  |
|  | **Married** | 0.602 (0.524-0.692) | <0.001 |  | 0.602 (0.558-0.649) | <0.001 |  | 0.433 (0.306-0.613) | <0.001 |  | 0.659 (0.568-0.765) | <0.001 |
| **Benign or borderline tumors history** | | |  |  |  |  |  |  |  |  |  |  |
|  | **No** | 1.000 [Reference] |  |  | 1.000 [Reference] |  |  | 1.000 [Reference] |  |  | 1.000 [Reference] |  |
|  | **Yes** | 0.735 (0.305-1.770) | 0.492 |  | 1.264 (0.865-1.847) | 0.225 |  | 1.455 (0.361-5.855) | 0.598 |  | 1.307 (0.621-2.750) | 0.481 |
| **Lateral** | |  |  |  |  |  |  |  |  |  |  |  |
|  | **Right** | 1.000 [Reference] |  |  | 1.000 [Reference] |  |  | 1.000 [Reference] |  |  | 1.000 [Reference] |  |
|  | **Left** | 0.973 (0.863-1.096) | 0.650 |  | 1.003 (0.932-1.079) | 0.947 |  | 1.109 (0.779-1.333) | 0.14 |  | 0.988 (0.854-1.142) | 0.866 |
| **Tumor location** | |  | 0.171 |  |  | 0.059 |  |  | 0.075 |  |  | 0.620 |
|  | **Outer quadrant** | 1.000 [Reference] |  |  | 1.000 [Reference] |  |  | 1.000 [Reference] |  |  | 1.000 [Reference] |  |
|  | **Inner quadrant** | 0.804 (0.678-0.954) | 0.012 |  | 0.920 (0.832-1.018) | 0.106 |  | 0.733 (0.486-1.105) | 0.138 |  | 0.929 (0.762-1.133) | 0.467 |
|  | **Others** | 1.094 (0.960-1.248) | 0.179 |  | 1.085 (1.000-1.177) | 0.051 |  | 1.292 (0.963-1.731) | 0.086 |  | 1.044 (0.888-1.229) | 0.600 |
| **Histology (ICD-O-3) ^b^** | | | 0.877 |  |  | 0.165 |  |  | 0.270 |  |  | 0.991 |
|  | **NST** | 1.000 [Reference] |  |  | 1.000 [Reference] |  |  | 1.000 [Reference] |  |  | 1.000 [Reference] |  |
|  | **ILC** | 1.215 (0.994-1.485) | 0.058 |  | 0.946 (0.836-1.070) | 0.376 |  | 1.263 (0.818-1.951) | 0.292 |  | 0.769 (0.590-1.003) | 0.052 |
|  | **Favorable** | 0.956 (0.755-1.210) | 0.709 |  | 0.934 (0.777-1.123) | 0.468 |  | 0.441 (0.207-0.939) | 0.034 |  | 0.423 (0.249-0.718) | <0.001 |
|  | **metaplastic** | 2.438 (1.307-4.548) | 0.005 |  | 2.269 (1.583-3.252) | <0.001 |  | 4.638 (1.721-9.501 | 0.002 |  | 5.457 (3.457-8.614) | <0.001 |
|  | **others** | 0.830 (0.553-1.246) | 0.369 |  | 1.206 (0.914-1.592) | 0.186 |  | 0.510 (0.163-1.595) | 0.247 |  | 1.102 (0.622-1.950) | 0.740 |
| **Grade** | |  | <0.001 |  |  | <0.001 |  |  | <0.001 |  |  | <0.001 |
|  | **Well** | 1.000 [Reference] |  |  | 1.000 [Reference] |  |  | 1.000 [Reference] |  |  | 1.000 [Reference] |  |
|  | **Moderately** | 1.265 (1.099-1.457) | 0.001 |  | 1.240 (1.131-1.359) | <0.001 |  | 1.623 (1.111-2.371) | 0.012 |  | 1.755 (1.392-2.212) | <0.001 |
|  | **Poorly** | 2.151 (1.830-2.529) | <0.001 |  | 2.017 (1.826-2.227) | <0.001 |  | 5.626 (3.882-8.154) | <0.001 |  | 5.890 (4.718-7.353) | <0.001 |
| **Subtype** | |  | <0.001 |  |  | <0.001 |  |  | <0.001 |  |  | <0.001 |
|  | **HR+/HER2-** | 1.000 [Reference] |  |  | 1.000 [Reference] |  |  | 1.000 [Reference] |  |  | 1.000 [Reference] | <0.001 |
|  | **HR+/HER2+** | 1.530 (1.217-1.924) | <0.001 |  | 1.381 (1.198-1.593) | <0.001 |  | 1.993 (1.205-3.300) | 0.007 |  | 2.167 (1.673-2.807) | <0.001 |
|  | **HR-/HER2-** | 1.908 (1.570-2.320) | <0.001 |  | 1.886 (1.694-2.100) | <0.001 |  | 4.828 (3.476-6.724) | <0.001 |  | 4.333 (3.645-5.151) | <0.001 |
|  | **HR-/HER2+** | 1.290 (0.860-1.936) | 0.218 |  | 1.560 (1.263-1.926) | <0.001 |  | 3.649 (1.945-6.743) | <0.001 |  | 3.530 (2.563-4.862) | <0.001 |
| **T** | |  |  |  |  |  |  |  |  |  |  |  |
|  | **T1** | 1.000 [Reference] |  |  | 1.000 [Reference] |  |  | 1.000 [Reference] |  |  | 1.000 [Reference] |  |
|  | **T2** | 2.560 (2.270-2.886) | <0.001 |  | 2.025 (1.877-2.185) | <0.001 |  | 4.305 (3.276-5.657) | <0.001 |  | 3.827 (3.308-4.427) | <0.001 |
| **N** | |  |  |  |  |  |  |  |  |  |  |  |
|  | **N0** | 1.000 [Reference] |  |  | 1.000 [Reference] |  |  | 1.000 [Reference] |  |  | 1.000 [Reference] |  |
|  | **N1** | 1.300 (0.739-2.297) | 0.366 |  | 1.708 (1.542-1.891) | <0.001 |  | 2.750 (1.132-6.679) | 0.026 |  | 3.023 (2.553-3.681) | <0.001 |
| **Breast surgery type** | |  |  |  |  |  |  |  |  |  |  |  |
|  | **BCS** | 1.000 [Reference] |  |  | 1.000 [Reference] |  |  | 1.000 [Reference] |  |  | 1.000 [Reference] |  |
|  | **Mastectomy** | 1.437 (1.225-1.687) | <0.001 |  | 1.736 (1.609-1.873) | <0.001 |  | 1.601 (1.132-2.266) | 0.008 |  | 2.049 (1.767-2.377) | <0.001 |
| **Radiation** | |  |  |  |  |  |  |  |  |  |  |  |
|  | **No/unknown** | 1.000 [Reference] |  |  | 1.000 [Reference] |  |  | 1.000 [Reference] |  |  | 1.000 [Reference] |  |
|  | **Yes** | 0.449 (0.382-0.528) | <0.001 |  | 0.498 (0.462-0.547) | <0.001 |  | 0.440 (0.303-0.637) | <0.001 |  | 0.486 (0.419-0.565) | <0.001 |
| **Chemotherapy** | |  |  |  |  |  |  |  |  |  |  |  |
|  | **No/unknown** | 1.000 [Reference] |  |  | 1.000 [Reference] |  |  | 1.000 [Reference] |  |  | 1.000 [Reference] |  |
|  | **Yes** | 0.513 (0.326-0.809) | 0.004 |  | 0.949 (0.844-1.067) | 0.382 |  | 1.131 (0.558-2.293) | 0.732 |  | 2.202 (1.849-2.623) | <0.001 |
| Note: AIA American Indian/Alaska Native; API Asian or Pacific Islander; NST no special type; ILC invasive lobular carcinoma; SLNB sentinel lymph node biopsy; BCS Breast reserving mastectomy/Lumpectomy; OS overall survival; BCSS, breast cancer specific survival. | | | | | | | | | | | | |

**Supplementary Table 5 C-index for internal and external validation**

| Model | C-index (95% CI) | |
| --- | --- | --- |
|  | Internal Validation | External Validation |
| OS for patients with SLNB | 0.698 (0.693-0.703) | 0.687 (0.678-0.696) |
| OS for patients without SLNB | 0.769 (0.760-0.779) | 0.769 (0.760-0.779) |
| BCSS for patients with SLNB | 0.695 (0.687-0.703) | 0.711 (0.697-0.725) |
| BCSS for patients without SLNB | 0.785 (0.769-0.800) | 0.820 (0.796-0.845) |

**Supplementary Table 6** The survival outcomes predicted by separate nomograms for the case used as an example

|  | **SLNB** | |  | **No SLNB** | |
| --- | --- | --- | --- | --- | --- |
|  | **OS** | **BCSS** |  | **OS** | **BCSS** |
| **Age：**75-79 | 30 | 25 |  | 32 | 15 |
| **Marital：**married | 0 | 0 |  | 0 | 0 |
| **Grade:** well, I | 0 | 0 |  | 0 | 0 |
| **Subtype:** HR-/HER2+ | 8 | 48 |  | 6 | 23 |
| **T:** T1 | 0 | 0 |  | 0 | 0 |
| **Radiation:** Yes | 0 | 0 |  | 0 | 0 |
| **Total points** | 38 | 73 |  | 38 | 38 |
| **3-year survival** | >95% | >95% |  | >90% | >95% |
| **5-year survival** | >90% | >95% |  | 87% | >95% |

**1.2 Supplementary Figures**

**Supplementary Figure1** The distribution of propensity score for matched and unmatched patients


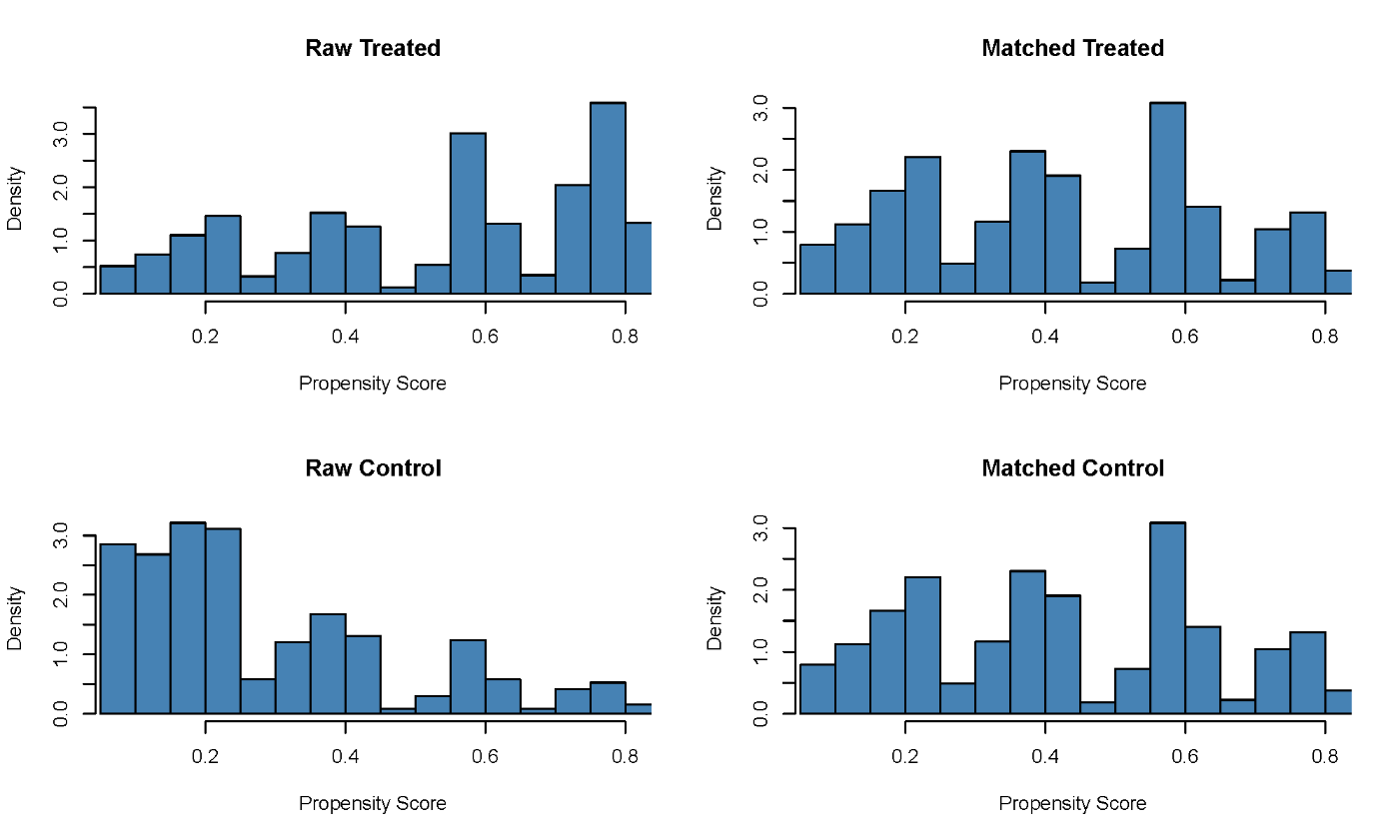

Supplement: Supplementary file 1 [file Data_Sheet_1.docx]
